# Corrigendum to ‘Impact of accumulative smoking exposure and chronic obstructive pulmonary disease on COVID-19 outcomes: report based on findings from the Japan COVID-19 task force’ [IJID 128 (2023) 121–127]

**DOI:** 10.1016/j.ijid.2024.107322

**Published:** 2025-02

**Authors:** Mayuko Watase, Katsunori Masaki, Shotaro Chubachi, Ho Namkoong, Hiromu Tanaka, Ho Lee, Takahiro Fukushima, Shiro Otake, Kensuke Nakagawara, Tatsuya Kusumoto, Takanori Asakura, Hirofumi Kamata, Makoto Ishii, Naoki Hasegawa, Yoshitaka Oyamada, Norihiro Harada, Tetsuya Ueda, Soichiro Ueda, Takashi Ishiguro, Ken Arimura, Fukuki Saito, Takashi Yoshiyama, Yasushi Nakano, Yoshikazu Mutoh, Yusuke Suzuki, Ryuya Edahiro, Hirohito Sano, Yasunori Sato, Yukinori Okada, Ryuji Koike, Yuko Kitagawa, Katsushi Tokunaga, Akinori Kimura, Seiya Imoto, Satoru Miyano, Seishi Ogawa, Takanori Kanai, Koichi Fukunaga, the Japan COVID-19 Task Force

**Affiliations:** 1 Division of Pulmonary Medicine, Department of Medicine, Keio University School of Medicine, Tokyo, Japan; 2 Department of Respiratory Medicine, National Hospital Organization Tokyo Medical Center, Tokyo, Japan; 3 Department of Infectious Diseases, Keio University School of Medicine, Tokyo, Japan; 4 Department of Pulmonary Medicine, Saitama City Hospital, Saitama, Japan; 5 Department of Respiratory Medicine, Juntendo University Faculty of Medicine and Graduate School of Medicine, Tokyo, Japan; 6 Department of Respiratory Medicine, Osaka Saiseikai Nakatsu Hospital, Osaka, Japan; 7 Department of Internal Medicine, JCHO (Japan Community Health Care Organization) Saitama Medical Center, Saitama, Japan; 8 Department of Respiratory Medicine, Saitama Cardiovascular and Respiratory Center, Kumagaya, Japan; 9 Department of Respiratory Medicine, Tokyo Women's Medical University, Tokyo, Japan; 10 Department of Emergency and Critical Care Medicine, Kansai Medical University General Medical Center, Moriguchi, Japan; 11 Respiratory Disease Center, Fukujuji Hospital, Japan Anti-Tuberculosis Association, Tokyo, Japan; 12 Department of Internal Medicine, Kawasaki Municipal Ida Hospital, Kawasaki, Japan; 13 Department of Infectious Diseases, Tosei General Hospital, Seto, Japan; 14 Department of Respiratory Medicine, Kitasato University, Kitasato Institute Hospital, Tokyo, Japan; 15 Department of Statistical Genetics, Osaka University Graduate School of Medicine, Suita, Japan; 16 Department of Respiratory Medicine, Tohoku University Graduate School of Medicine, Sendai, Japan; 17 Department of Preventive Medicine and Public Health, Keio University School of Medicine, Tokyo, Japan; 18 Integrated Frontier Research for Medical Science Division, Institute for Open and Transdisciplinary Research Initiatives, Osaka University, Suita, Japan; 19 The Center for Infectious Disease Education and Research (CiDER), Osaka University, Suita, Japan; 20 Laboratory of Statistical Immunology, Immunology Frontier Research Center (WPI-IFReC), Osaka University, Suita, Japan; 21 Department of Genome Informatics, Graduate School of Medicine, the University of Tokyo, Tokyo, Japan; 22 Laboratory for Systems Genetics, RIKEN Center for Integrative Medical Sciences, Kanagawa, Japan; 23 Medical Innovation Promotion Center, Tokyo Medical and Dental University, Tokyo, Japan; 24 Department of Surgery, Keio University School of Medicine, Tokyo, Japan; 25 Genome Medical Science Project (Toyama), National Center for Global Health and Medicine, Tokyo, Japan; 26 Institute of Research, Tokyo Medical and Dental University, Tokyo, Japan; 27 Division of Health Medical Intelligence, Human Genome Center, the Institute of Medical Science, the University of Tokyo, Tokyo, Japan; 28 M&D Data Science Center, Tokyo Medical and Dental University, Tokyo, Japan; 29 Department of Pathology and Tumor Biology, Kyoto University, Kyoto, Japan; 30 Institute for the Advanced Study of Human Biology (WPI-ASHBi), Kyoto University, Kyoto, Japan; 31 Department of Medicine, Center for Hematology and Regenerative Medicine, Karolinska Institute, Stockholm, Sweden; 32 Division of Gastroenterology and Hepatology, Department of Medicine, Keio University School of Medicine, Tokyo, Japan

The authors would like to point out that the caption for Figure 2 in the published version of their paper contained an error. The correct caption is below:

Figure 2 legend (corrected version):

(a) Incidence of IMV among non-COPD patients classified by PY and COPD patients.

(b) Results of the univariate analysis of need for IMV on all smoker patients (b-1), smoker patients aged 65 years or younger (b-2), and smoker patients over 65 years old (b-3).

**p< 0.001, *p<0.05. IMV, invasive mechanical ventilation; COPD, chronic obstructive pulmonary disease; PY, pack-year.

